# Dietary Gut Microbial Metabolites, Short-chain Fatty Acids, and Host Metabolic Regulation

**DOI:** 10.3390/nu7042839

**Published:** 2015-04-14

**Authors:** Mayu Kasubuchi, Sae Hasegawa, Takero Hiramatsu, Atsuhiko Ichimura, Ikuo Kimura

**Affiliations:** 1 Department of Applied Biological Science, Graduate School of Agriculture, Tokyo University of Agriculture and Technology, Fuchu-shi, Tokyo 183-8509, Japan; E-Mails: 50012152019@st.tuat.ac.jp (M.K.); 50012152057@st.tuat.ac.jp (S.H.); 2 Department of Genetic Biochemistry, Kyoto University Graduate School of Pharmaceutical Science, Sakyo, Kyoto 606-8501, Japan; E-Mail: hiramatsu.takerou.88v@st.kyoto-u.ac.jp; 3 Department of Pharmacogenomics, Kyoto University Graduate School of Pharmaceutical Science, Sakyo, Kyoto 606-8501, Japan; E-Mail: ichimura.atsuhiko.2r@kyoto-u.ac.jp

**Keywords:** SCFA, gut microbiota, energy metabolism, GPR41, GPR43

## Abstract

During feeding, the gut microbiota contributes to the host energy acquisition and metabolic regulation thereby influencing the development of metabolic disorders such as obesity and diabetes. Short-chain fatty acids (SCFAs) such as acetate, butyrate, and propionate, which are produced by gut microbial fermentation of dietary fiber, are recognized as essential host energy sources and act as signal transduction molecules via G-protein coupled receptors (FFAR2, FFAR3, OLFR78, GPR109A) and as epigenetic regulators of gene expression by the inhibition of histone deacetylase (HDAC). Recent evidence suggests that dietary fiber and the gut microbial-derived SCFAs exert multiple beneficial effects on the host energy metabolism not only by improving the intestinal environment, but also by directly affecting various host peripheral tissues. In this review, we summarize the roles of gut microbial SCFAs in the host energy regulation and present an overview of the current understanding of its physiological functions.

## 1. Introduction

Diet is a most important factor for daily nutrient acquisition. However, the dysregulation of energy homeostasis by excessive intake leads to obesity. Obesity is currently one of the most serious public health challenges worldwide, especially in the Western world, because of its increasing prevalence and its contribution to a complex of symptoms collectively called the “metabolic syndrome” [1,2]. The progress of obesity is caused by a long-term imbalance between energy intake and expenditure, which in turn influences multiple effector pathways involving metabolites and hormones [3]. Excessive food intake, especially of high-fat and sugar products, together with insufficient exercise and genetic susceptibility, are considered risk factors for developing obesity. Recent research has demonstrated that changes in the gut microbiota are closely linked with metabolic disorders such as obesity and type 2 diabetes [4,5,6]. One of the most important roles of the gut microbiota is to catabolize dietary fibers that are not completely hydrolyzed by the host enzymes during digestion [7]. The main products of intestinal bacterial fermentation of dietary fiber are short-chain fatty acids (SCFAs) such as acetate, propionate, and butyrate [8]. SCFAs can be used for *de novo* synthesis of lipids and glucose, which are the main energy sources for the host [9].

## 2. Gut Microbial Composition and Metabolic Disease

The gut microbiota is a complex of microorganisms including more than 100 trillion cells of 400 species, which is equivalent to ten times the total number of cells in the human body [10]. Because the majority of the gut microbes are strictly anaerobic their identification and functional analysis has been difficult. However, since the advent of metagenomics, it has been revealed that gut microbes play an important role in host metabolic and immune homeostasis. Recent studies indicate that the gut microbiota can be considered an environmental factor that affects host adiposity and can contribute to obesity [11,12]. The human microbiome contains 150 times more genes than the human genome [13]. Metagenomic analysis of the gut microbiome in obese mice and humans indicated that expression of genes involved in carbohydrate metabolism predominated. Transplantation of the microbiota from obese mice into the gut of germ-free mice significantly increased adiposity in the recipient mice in comparison with transplantation of a lean microbiota. Moreover, cohort studies in Europe and China revealed that, despite the ethnic and dietary differences patients with type 2 diabetes had a lower proportion of butyrate-producing and a larger proportion of non-butyrate-producing Clostridiales [14,15]. In addition, although the functions encoded by the metagenomes of butyrate-producing bacteria were comparable between European and Chinese, the prevalent bacterial taxa were markedly different in the two cohorts. This indicates that the gut microbiota is notably affected by diet and ethnicity. Roux-en-Y gastric bypass (RYGB) induces dramatic and sustained weight loss by restricting the amount of food that can be ingested, thereby improving insulin sensitivity and type 2 diabetes. RYGB is currently the most effective treatment for obesity. The resulting metabolic improvement cannot be explained by the reduced calorie intake alone; the altered gut physiology following RYGB contributes to an altered intestinal microbial ecology in mice, rats, and humans, which may contribute to the improved host metabolism. Liou *et al.* reported that the change in the gut microbiota upon transplantation of RYGB-related fecal microbiota directly contributed to reduced weight and adiposity [16]. Therefore, the gut microbiota is considered an environmental factor that modulates the host metabolism and may contribute to metabolic disorder.

## 3. Metabolic Beneficial Effects by SCFAs

Recently, dietary fibers have gained increasing interest because they exert beneficial effects on metabolic functions such as body weight, food intake, glucose homeostasis, and insulin sensitivity [17,18]. Hence, dietary fiber intake reduces risk of inflammatory bowel disease, cardiovascular disease, colon cancer, obesity and diabetes [19,20]. In the last few decades, it has been hypothesized that SCFAs might play a key role in the prevention and treatment of metabolic syndrome, bowel disorders, and cancer [21,22,23]. With regard to the energy metabolism, butyrate improved insulin sensitivity and increased the energy expenditure in dietary-obese mice [24]. Butyrate and propionate were shown to protect against diet-induced obesity and regulated the gut hormones [25]. The oral administration of acetate improved glucose tolerance and suppresses obesity [26]. Clinical studies showed that the administration of SCFAs has a positive effect on the treatment of ulcerative colitis, Crohn's disease, and antibiotic-associated diarrhea and obesity [22,27,28,29]. In obese subjects, propionate significantly increased the release of postprandial plasma peptide YY (PYY) and glucagon-like peptide-1 (GLP-1) from colonic cells, and reduced the energy intake. Inulin-propionate ester administrated at 10 g per day over a period of 24 weeks significantly reduced weight gain, intra-abdominal adipose tissue distribution and intrahepatocellular lipid content, and improved insulin resistance in the inulin control group [29]. On the other hand, SCFAs also utilize as host energy source, therefore SCFAs are regarded as cause of increasing energy harvest from diet, linked to the obese phenotype by changes of gut microbiota composition. To clarify these controversial, the molecular mechanisms of SCFAs was next investigated.

## 4. Molecular Mechanisms Involved in Host Metabolic Regulation by SCFAs

As gut microbial SCFAs have been reported to exert beneficial effects on the host metabolism, in this paragraph we focus on the elucidation of the molecular mechanisms involved. Total SCFAs in gut lumen is ~100 mM, in blood is, portal ~400 μM and peripheral ~100 μM [30]. Hence, SCFAs exhibit the tissue specific physiological function by this concentration gradient. Butyrate enhances fatty acid oxidation and thermogenesis by increasing the expression of peroxisome proliferator-activated receptor-gamma coactivator-1α (PGC-1α) and the phosphorylation of adenosine-monophosphate-activated kinase (AMPK) in muscle and liver tissues, and the expression of PGC-1α and mitochondrial uncoupling protein-1 (UCP-1) in brown adipose tissues [21]. Propionate and butyrate activate intestinal gluconeogenesis via a gut-brain neural circuit, thereby promoting metabolic benefits on body weight and glucose control [31]. Acetate reduces the appetite by changing the expression profiles of appetite regulatory neuropeptides in the hypothalamus through activation of TCA cycle [32]. Various studies that had been focused on the elucidation of the SCFA target molecules in the host demonstrated that G-protein-coupled receptors (GPCR) act as SCFA receptors [33,34].

## 5. SCFA Receptors

### 5.1. FFAR2/GPR43

GPR43, also known as FFAR2 has been identified as an SCFA receptor and is mainly activated by acetate and propionate followed by butyrate [35,36]. FFAR2 is expressed in intestinal endocrine L-cells, where it stimulates the release of PYY and GLP-1. SCFA-induced FFAR2 activation was shown to promote the secretion of GLP-1 in mouse colonic primary cultures and in enteroendocrine STC-1 cells [37]. Ffar2-deficient mice exhibited decreased SCFA-induced secretion of GLP-1 both *in vitro* and *in vivo* and improved insulin resistance. FFAR2 is abundantly expressed in adipose tissues as well. FFAR*2* expression was significantly increased in white adipose tissues of high-fat-diet-induced obese mice in comparison with normal chow-fed mice [38]. In addition, SCFAs inhibited isoproterenol-induced lipolysis in a concentration-dependent manner in mouse 3T3-L1-derived adipocytes [38]. Ge *et al.* later demonstrated that this effect was dependent on FFAR2 using Ffar2-deficient mice [39]. Moreover, in white adipose tissues, FFAR2 activation by SCFAs suppressed adipose-specific insulin signaling, leading to the inhibition of fat accumulation [40]. Ffar2-deficient mice were shown to exhibit obesity, whereas mice overexpressing adipose-specific Ffar2 overexpressed mice exhibited leanness under normal conditions. However, these mouse strains did not exhibit theses respective phenotypes when grown under germ-free conditions or when treated with antibiotics. This indicated that the source of FFAR2 ligands was dependent on gut microbes and that FFAR2 regulates adipose-insulin signaling by sensing microbial SCFAs, thereby regulating fat accumulation and maintaining body energy homeostasis. Hence, FFAR2 activation by SCFAs promotes GLP-1 secretion in the gut and suppression of fat accumulation in adipose tissue, leading to increased insulin sensitivity. FFAR2 is also expressed in immune tissues. Gut microbiota and FFAR2 regulate inflammatory responses in colitis [41]. In addition, SCFAs regulate the size and function of the colonic pool of regulatory T cells and protect against colitis in a FFAR2-dependent manner in mice [42]. Since the inflammatory response is also related to the development of obesity and type 2 diabetes, the regulation of immune function via FFAR2 may be also related to the metabolic beneficial effects by SCFAs.

### 5.2. FFAR3/GPR41

FFAR3 has also been identified as an SCFA receptor. However, the ligand affinity to SCFAs differs between FFAR2 and FFAR3; FFAR3 is activated mainly by propionate and butyrate [35,36]. Like FFAR2, FFAR3 is also expressed in PYY- and GLP-1-secreting endocrine L-cells, indicating its involvement in energy homeostasis [43]. The secretion of PYY and GLP-1 was shown to be reduced in primary cultured endocrine cells derived from Ffar3-deficient mice [37,44]. After transplantation of specific microbes to gnotobiotic mice, wild-type but not Ffar3-deficient mice showed an increase in PYY levels. Therefore, the secretion of PYY from intestinal L-cells is due to activation of FFAR3 by SCFAs produced by the gut microbes. FFAR3 is abundantly expressed in sympathetic ganglia as well [45]. FFAR3 activation by propionate increases the heart rate and energy expenditure through sympathetic activation. In addition, sympathetic activation by FFAR3 directly leads to noradrenalin release from the sympathetic neurons [46]. This indicates that FFAR3 regulates sympathetic activity by sensing the nutritional state, thereby maintaining body energy homeostasis. It has been reported that FFAR3 contributes to the improvement of insulin resistance by dietary fibers through activation of FFAR3 expressed in the peripheral nerves by SCFAs produced by gut microbes [30]. This implies that FFAR3 stimulation by SCFAs exhibits beneficial effects on the host metabolism via the peripheral nervous system and hormone secretion in the gut. Similar to FFAR2, FFAR3 affects the inflammatory response. Propionate was shown to affect bone marrow hematopoiesis in an FFAR3-dependent manner in mice, by inducing an enhanced generation of macrophage and dendritic cell precursors thereby influencing the allergic inflammatory response in airway disease via FFAR3 [47]. Therefore, FFAR3 may be involved in the beneficial effects of SCFAs on host metabolism through the regulation of immune responses.

### 5.3. Other SCFA Receptors (GPR109A and OLFR78)

GPR109A was first identified as a receptor for niacin and is also activated by β-hydroxybutryate and butyrate, but not by acetate and propionate [47]. The EC50 value for GPR109A activation by butyrate is approximately 1mM [47]. GPR109A is expressed in the epithelial cells of the colon and expression decreases in the absence of the gut microbiota in germ-free mice [48]. GPR109A activation by butyrate suppresses colonic inflammation and carcinogenesis by promoting anti-inflammatory properties in colonic macrophages and dendritic cells, which induce the differentiation of regulatory and IL10-producing T cells [49]. In addition, GPR109A is expressed in adipose tissues and activated adipose tissue macrophages where it regulates lipid homeostasis [47,50,51,52]. Olfactory receptor 78 (OLFR78) was identified as SCFAs in a ligand screen for orphan GPCRs [53]. OLFR78 is activated by acetate and propionate but not by butyrate; the EC50 value are 2.35mM and 920 μM, respectively. Olfr78 is expressed in blood vessels, especially localize in renal vessel where it is involved in renin secretion. The binding of gut microbiota-derived SCFAs to OLFR78 induces the release of renin, which is involved in the modulation of the blood pressure [53,54,55]. The metabolic effects of this SCFAs receptor are unclear to date. It is expected that further analysis will elucidate its function in energy metabolism.

## 6. Epigenetic Regulation by SCFAs

Gene expression is regulated by the modulation of histone acetylation by histone acetyltransferases and histone deacetylases (HDAC). SCFAs produced by gut microbes inhibit histone deacetylase activity and consequently modulate gene expression. Butyrate is the most potent inhibitor of HDAC, with a maximum efficiency of approximately 80% inhibition of HDAC1/2, and its *in vitro* Ki value is approximately 58 μM [56]. Propionate has a maximum inhibitory efficiently of approximately 60%. SCFAs function as non-competitive inhibitors of HDACs. It has been shown that butyrate and propionate selectively inhibit HDAC1 and HDAC3. With regard to the influence of the inhibition of HDAC by SCFAs on the host physiological functions, it has been reported that propionate and butyrate produced by gut microbes promote the generation of peripheral regulatory T-cells [57,58]. Gut microbe-derived butyrate induces the differentiation of colonic regulatory T cells by enhancing histone H3 acetylation in the promoter and conserved non-coding sequence regions of the Foxp3 locus, and reduces the development of colitis [58]. In addition, the putative link between the microbiota and epigenetic regulation has been examined in obese and type 2 diabetes patients as compared to lean control subjects [59]. The diversity of the gut microbiota and the degree of methylation of the FFAR3 promoter region were significantly lower in the obese and type 2 diabetic patients compared to lean individuals, demonstrating a correlation between a higher body mass index and lower methylation of FFAR3 [59]. Therefore, epigenetic regulation may be also related to the beneficial effects by SCFAs on host metabolism.

## 7. Conclusions

**Figure 1:**
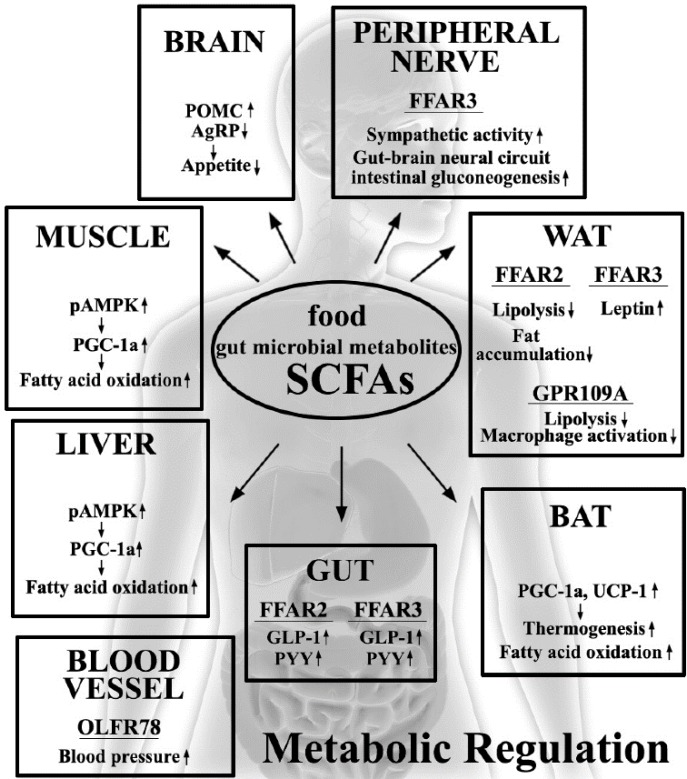
Effects of dietary gut microbial short-chain fatty acids (SCFAs) on the regulation of host metabolic functions. SCFAs affect host homeostasis through the stimulation of SCFA receptors, FFAR2, FFAR3, GPR109A and OLFR78.

In recent years, various host metabolic and immune response pathways have been reported to be related to diet and gut microbiota. Several studies have provided evidence that SCFAs, which are the end products of the fermentation of dietary fibers by the anaerobic intestinal microbiota, have beneficial effects on the host energy metabolism and inflammatory responses, because the host SCFA receptors and target molecules are expressed in both metabolic and immune tissues (Figure 1). As the inflammatory response is related to the development of obesity and type 2 diabetes, the SCFAs and their target molecules constitute potential therapeutic targets for the treatment of these diseases. Although the exact signaling mechanisms and physiological functions of SCFAs in the host peripheral tissues are unclear, a deeper understanding of the regulation of the energy metabolism and the inflammatory responses by the dietary SCFAs represents an important avenue for research in medicine and drug development for the prevention and treatment of obesity and type 2 diabetes.
